# Speech Intelligibility and Spatial Release From Masking Improvements Using Spatial Noise Reduction Algorithms in Bimodal Cochlear Implant Users

**DOI:** 10.1177/23312165211005931

**Published:** 2021-04-29

**Authors:** Ayham Zedan, Tim Jürgens, Ben Williges, Birger Kollmeier, Konstantin Wiebe, Julio Galindo, Thomas Wesarg

**Affiliations:** 1 Medizinische Physik und Exzellenzcluster “Hearing4all,” Carl-von-Ossietzky Universität Oldenburg, Oldenburg, Germany; 2 Institut für Akustik, Technische Hochschule Lübeck, Lübeck, Germany; 3 Department of Clinical Neurosciences, University of Cambridge, Cambridge, United Kingdom; 4 Department of Otorhinolaryngology – Head and Neck Surgery, Faculty of Medicine, Medical Center – University of Freiburg, University of Freiburg, Freiburg, Germany

**Keywords:** bimodal, cochlear implant, virtual acoustics, speech intelligibility, spatial noise reduction algorithms

## Abstract

This study investigated the speech intelligibility benefit of using two different spatial noise reduction algorithms in cochlear implant (CI) users who use a hearing aid (HA) on the contralateral side (bimodal CI users). The study controlled for head movements by using head-related impulse responses to simulate a realistic cafeteria scenario and controlled for HA and CI manufacturer differences by using the master hearing aid platform (MHA) to apply both hearing loss compensation and the noise reduction algorithms (beamformers). Ten bimodal CI users with moderate to severe hearing loss contralateral to their CI participated in the study, and data from nine listeners were included in the data analysis. The beamformers evaluated were the adaptive differential microphones (ADM) implemented independently on each side of the listener and the (binaurally implemented) minimum variance distortionless response (MVDR). For frontal speech and stationary noise from either left or right, an improvement (reduction) of the speech reception threshold of 5.4 dB and 5.5 dB was observed using the ADM, and 6.4 dB and 7.0 dB using the MVDR, respectively. As expected, no improvement was observed for either algorithm for colocated speech and noise. In a 20-talker babble noise scenario, the benefit observed was 3.5 dB for ADM and 7.5 dB for MVDR. The binaural MVDR algorithm outperformed the bilaterally applied monaural ADM. These results encourage the use of beamformer algorithms such as the ADM and MVDR by bimodal CI users in everyday life scenarios.

Cochlear implants (CIs) enable many of their users to achieve good speech intelligibility scores in quiet which can reach normal performance in sentence recognition tests (Gifford et al., 2018). However, CI users struggle to understand speech in background noise and complex auditory scenarios with reverberation (Poissant et al., 2006; Whitmal & Poissant, 2009) and several noise sources (Weissgerber et al., 2017). CI users with residual hearing at the contralateral side of the CI demonstrate better speech intelligibility in noise when their CI is supplemented with a contralateral hearing aid (HA; e.g., Ching et al., 2006). Those who use an HA on the contralateral side are referred to as bimodal CI users. Consequently, the usage of both devices is strongly encouraged whenever possible (Gifford et al., 2015). It is well known that the CI provides access to high frequencies and temporal envelope cues, while the HA complements this by providing acoustic fine-structure cues in the other ear, mainly at low frequencies (Gifford et al., 2015; Seeber et al., 2004; Yoon et al., 2015). However, the exact mechanism by which bimodal CI users combine those streams of information is still unclear. A recent study on speech intelligibility in noise by bimodal CI users with a wide range of hearing loss on the HA side (Kokkinakis & Pak, 2014; Williges et al., 2019) suggests that these listeners rely on ‘scenario dependent better ear listening.’ This means that for a given acoustic scenario, the bimodal CI users focus automatically on the side with the respective better-performing ear.

Therefore, an obvious way to improve the speech intelligibility of bimodal CI users is to leverage the CI and HA devices to improve the quality of the speech signal delivered to the binaural auditory system. This is achieved by using noise reduction algorithms that aim to increase the signal-to-noise ratio (SNR) of the input audio signal. In principle, noise reduction algorithms operate by separating noise from the target signal either in the spectral or spatial domain (Kokkinakis et al., 2012). Single-channel noise reduction algorithms attenuate noise by using spectral and statistical properties of speech and noise (Boll, 1979). Beamformers, that is, spatial noise reduction algorithms, create a spatial attenuation pattern that depends on the sound incident angle, characterized by the directivity index (Kollmeier et al., 1993; Van Veen & Buckley, 1988). The word *beam* refers to a small subset of angles that correspond to the lowest attenuation which should be directed toward the signal of interest. The directivity index of a beamformer is determined by the method in which signals of multiple microphones are combined to utilize the constructive and destructive interference property of sound (Stadler & Rabinowitz, 1993). Spatial noise reduction algorithms have been shown to significantly improve speech intelligibility with all kinds of CI and HA users. That includes unilateral CI users (Mosnier et al., 2017), unilateral CI users that use contralateral routing of signals (Kurien et al., 2019), bilateral CI users (Baumgärtel et al., 2015a), and bimodal CI users (Buechner et al., 2014; Devocht et al., 2016; Ernst et al., 2019; Vroegop et al., 2018; Weissgerber et al., 2017).

Head movements and orientation were suggested to have a moderating effect on the benefit of beamformers, for instance, as was pointed out in Ernst et al. (2019). They found differences in SNR improvement between a dummy head and their subjects measured in free field, which they attributed to the algorithms not adapting fast enough to head movements. The aforementioned studies assessing the benefit of beamformers in bimodal CI users were conducted in free-field conditions, where subjects were able to freely move their heads. Furthermore, in more realistic acoustic environments, Grange and Culling (2016) have shown that even small amounts of head orientation differences can result in considerable SNR changes at the hearing device’s microphones. Moreover, Hendrikse et al. (2020) investigated the effect of head movements on algorithm benefit in various spatial acoustic scenarios, and they found a significant detrimental effect on the benefit provided by adaptive beamformers. Consequently, the influence of the subject’s head orientation and movement on the beamformers’ benefits reported by free-field studies cannot be ruled out. However, head movements can be controlled for by simulating an acoustic scenario using head-related impulse responses (HRIRs) and directly delivering the signal to an in-ear headphone for the acoustic ear and direct audio input (DAI) for the CI ear, as was done in Baumgärtel et al. (2015a) for bilateral CI users, and in Völker et al. (2015) for bilateral HA users. The present study extends the work of these two studies by measuring the effect of beamformers in bimodal CI users while excluding secondary effects and strictly controlling for the influence of as many parameters as possible:

- Head movements were controlled for by simulating a realistic cafeteria scenario using virtual acoustics with HRIRs and delivering the resulting audio signals via the DAI cable to the CI and an insert earphone to the HA ear.
- Algorithm implementations and HA fitting parameters were controlled for by using the same master hearing aid (MHA, Grimm et al., 2006) implementation for all participants.
- The algorithms were given time to adapt to the spatial scenario to evaluate speech reception thresholds (SRTs) and spatial release from masking (SRM) so that the comparison of the algorithms’ benefit was as isolated from adaptation as possible.
- In addition, differences due to CI devices were controlled for by replacing every subject’s own sound processor with a loaner CI sound processor.

In their work, Baumgärtel et al. (2015a) and Völker et al. (2015) evaluated the speech intelligibility benefit of using different noise reduction algorithms in bilateral CI and bilateral HA users, respectively, while controlling for the aforementioned secondary factors. They found that spatial noise reduction algorithms resulted in a considerably higher benefit than spectral noise reduction algorithms in different realistic scenarios. This article follows their work by evaluating two beamformers from their study, the adaptive differential microphone (ADM) and fixed minimum variance distortionless response (MVDR), but this time, in bimodal CI users. The ADM and MVDR are relatively simple and effective beamformers (Baumgärtel et al., 2015b). ADMs are mostly monaurally implemented and are widely used both in HAs and CIs of almost all manufacturers. An example of the ADM is the UltraZoom (Sonova, Stäfa, Switzerland; see Advanced Bionics, 2013; Buechner et al., 2014). Beam (Cochlear, Sydney, Australia; see Mauger et al., 2014) and the “Adaptive directional microphone mode” by MED-EL (Innsbruck, Austria; see De Ceulaer et al., 2019) which are similar in idea and implementation to the ADM. The MVDR used in this study was a fixed binaural beamformer designed to block diffuse noise sources. However, its binaural implementation, as with binaural algorithms in general, requires a link between the two devices because the signals across right and left devices need to be exchanged. This can be implemented as a wired solution (which can be inconvenient to the user) or as a wireless solution, introducing a transmission delay (latency) and higher power consumption (Dieudonné & Francart, 2018; Li et al., 2019), neither of those is a standard in the current CI technology. It is expected that binaural beamforming technology will see wider implementation in CIs, as first CI manufacturers employ low-latency wireless links (as, e.g., used in Ernst et al., 2019). Concerning performance, Baumgärtel et al. (2015a) found a larger benefit for using the MVDR over the ADM and that it was significantly higher in bilaterally implanted CI users when compared with normal-hearing subjects and bilateral HA users (Völker et al., 2015). Baumgärtel et al. (2015a) attributed the effect to the deterioration of binaural cues by the beamformer which normal-hearing subjects and HA users would usually benefit from in the absence of the beamformer. These subjects’ own binaural processing is based on the analysis of the acoustic temporal fine structure of the left and right ear signals, as proposed, for example, by Beutelmann and Brand (2006). While the MVDR provides an SNR improvement, it also distorts the binaural cues such that separation of speech in noise by the subject’s own binaural processing is strongly diminished, trading off binaural hearing with better-SNR monaural hearing. This showed that the benefit of using different beamforming algorithms may vary across different subject groups and individuals.

This study aimed to measure and compare the speech intelligibility benefit of using two types of beamformers for bimodal CI listeners while controlling for as many secondary factors as possible. The first type was a monaural beamformer, the ADM, implemented independently in each device. The second type was the binaurally implemented MVDR. The factors controlled for include algorithm implementations, HA fitting parameters, algorithm adaptation times, head movements, and CI and HA manufacturer differences. Furthermore, SRM was assessed.

## Materials and Methods

The subjects received reimbursement for travel and accommodation costs. Ethical approval was granted by the Medical Ethics commission of the University of Oldenburg (no. 097/2016) and the Ethics Committee of the University of Freiburg (no. 414/16). All study subjects signed informed participation consent.

### Participants

Subjects that satisfied the inclusion criteria according to their most recent entries in our clinical databases were recruited. Each subject had to have at least 1 year of experience with his/her CI and regular daily use of the HA on the contralateral side. Only subjects using Nucleus implants and sound processors (Cochlear Ltd, Sydney, Australia) were recruited, more specifically, the implant had to be compatible with Cochlear’s CP910 sound processor. Furthermore, air-conduction hearing thresholds in the HA aided ear of less than or equal to 80 dB HL at 500 Hz and 100 dB HL at 1 kHz were set as an inclusion criterion. Subjects with additional handicaps, for example, blindness, were not included.

**Figure 1.**
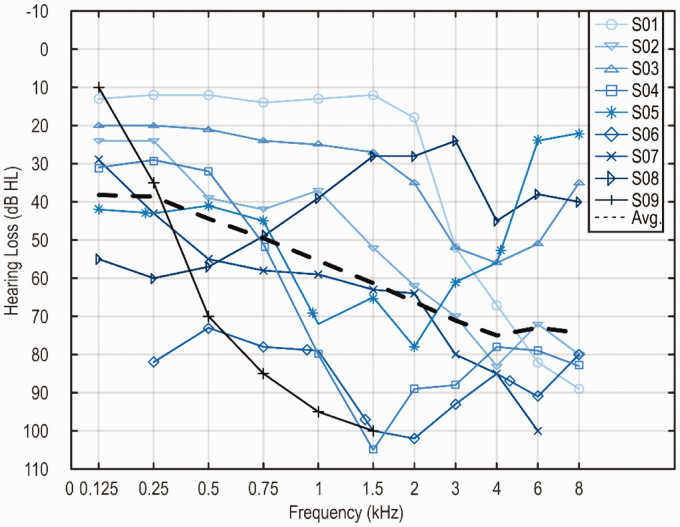
Air-Conduction Hearing Thresholds of the HA Side of the Nine Subjects.

Ten bimodal CI users, who were native German speakers, participated in this study. However, one subject had to be excluded from data analysis due to not fully complying with the inclusion criteria during testing. The age of the remaining nine subjects ranged from 20 to 69 years, with an average and standard deviation of 47.6 ± 18.6 years. Figure 1 shows the audiograms of the HA side of the nine subjects. The air-conduction hearing threshold of subject S06 exceeded 80 dB HL at 250 Hz; nevertheless, she had good speech understanding with her HA alone.

In addition, due to the asymmetry in the simulated acoustic scenario, described later, only subjects who had a CI on the right side and an HA on the left side were included. Subject S09 used a CI sound processor with electroacoustic hearing in the implanted ear. Note that the exchange of the subjects’ sound processor with the loaner CI processor with keeping the overlapping map meant that subject S09 did not have access to ipsilateral acoustic hearing during the experiment. Table 1 shows the subjects’ characteristics including age, etiology, implant, sound processor, duration of CI and HA usage, and monaural SRTs in noise obtained with the Oldenburg sentence test.

**Table 1.** Subjects’ Characteristics The text inside the parentheses belongs to the N.M. Abbreviation: see below.

Subj. ID	Age (years)	Biological gender	Etiology		Deafness duration (CI side, years)	Implant	CI use (months)	S0N0 SRT (dB SNR)	
			Left (HA) side	Right (CI) side				HA	CI
S01	63.8	Male	Progressive	Ménière’s disease	7.5	CI512	38	‒3.5	‒0.8
S02	67.2	Male	Progressive	Progressive, acute hearing loss	0.8	CI512	32	‒2.6	3.6
S03	50.2	Male	Progressive	Trauma	37.8	CI512	40	‒4.9	‒1.9
S04	69.3	Male	Progressive	Progressive	3.6	CI422	57	4.7	‒0.2
S05	24.1	Female	LVA syndrome	LVA syndrome	0.8	CI24RE CA	67	‒2.4	‒3.1
S06	20.9	Female	Hereditary	Hereditary	0.9	CI512	56	‒2.2	‒1.7
S07	55.6	Female	Endolymphatic hydrops	Ménière’s disease	0.4	CI512	23	0	0.1
S08	46.4	Male	Scarlet fever	Scarlet fever	39.8	CI512	53	‒2.2	N.M.
S09	29.5	Male	Ototoxic	Ototoxic	3.0	Hybrid L	134	‒2.1	‒3.8

*Note*. CI = cochlear implant; SRT= speech reception threshold; SNR = signal-to-noise ratio; HA = hearing aid; LVA = Large Vestibular Aqueduct; N.M. = not measurable, the CI user was not able to achieve 50% correct answers.

### Speech Recognition Testing

SRTs in noise, that is, SNRs in dB for 50% correct word recognition, were measured using the German matrix test, that is, the Oldenburg sentence test (OlSa, Wagener et al., 1999). Each sentence of the OlSa had a fixed structure: name-verb-number-adjective-object, for example, Peter hat drei grüne Autos (translated: Peter has three green cars). The sentences of the OlSa corpus are semantically correct but do not necessarily carry a meaningful message. A sentence was built by randomly picking 1 out of 10 possibilities for each of the five words. Two noise types were used in this study, a stationary noise with the same long-term spectrum as the OlSa speech material (“OlNoise”) or a 20-talker babble noise which consisted of 20 male speakers talking simultaneously (Baumgärtel et al., 2015b). The test started with an SNR of 0 dB by setting the speech and noise levels to an average of 65 dB sound pressure level (SPL) across all microphone channels (microphone configurations will be introduced in the following section). The SNR was then varied adaptively according to the A1 procedure described in Brand and Kollmeier (2002) by fixing the speech level at 65 dB SPL, a typical conversational speech level, and varying the noise level. Lists of 20 sentences were used.

### Simulated Cafeteria Scenario

The cafeteria spatial scenario simulated in this study had a relatively high reverberation time (T60) of 1.25 s and was realized by utilizing a virtual acoustics HRIR database from Kayser et al. (2009). The HRIRs used were recorded by Kayser et al. (2009). The database provided HRIRs of an in-ear microphone and three microphones on behind-the-ear HA cases fitted on each of the right and left ears of a KEMAR (Knowles Electronic Manikin for Acoustic Research) head and torso simulator (G.R.A.S, Holte, Denmark) in a real cafeteria. The HRIRs of the front and rear microphones on each side were used, while the HRIRs of the middle on-case microphones and in-ear microphones were not used. The speech and noise signals were convolved with the HRIR to obtain four simulated microphone signals. The frontal microphones were used to simulate the no-beamforming (NoBF) strategy, and both the frontal and rear microphones of the left and right sides were used as input to both the ADM and MVDR beamformers.

**Figure 2.**
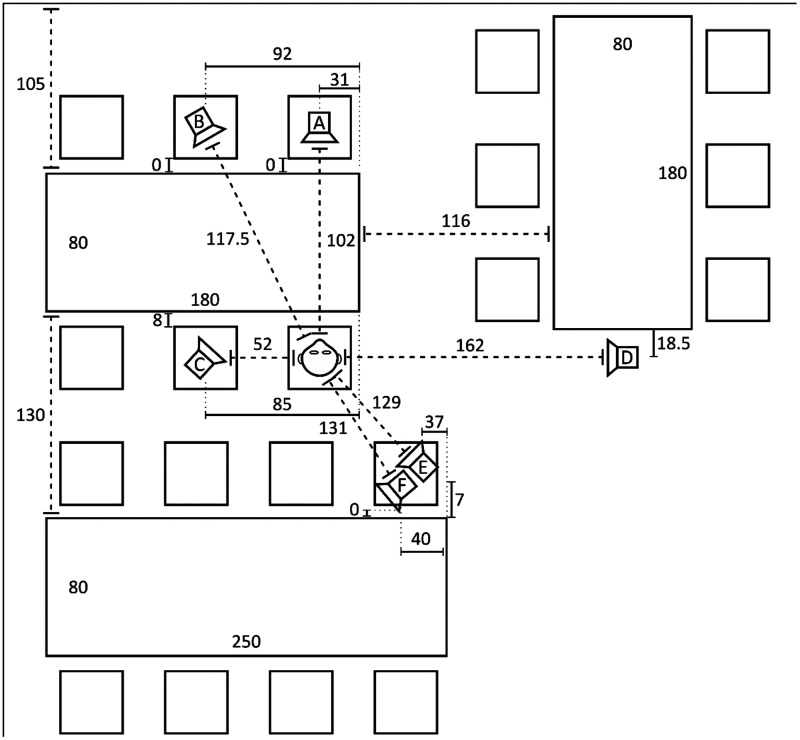
A Diagram of the Simulated Cafeteria Scenario. The head symbol represents the listener who is sitting at the table with face pointing toward Position A. The target speech was always presented from Position A, which is 102 cm away from the listener. In S_0_N_–90_, S_0_N_0_, and S_0_N_+90_, the noise was presented from Positions C, A, and D from 52 cm, 102 cm, and 162 cm, respectively. In the S_0_N_20TB_ scenario, 20-talker babble sources were evenly distributed over Positions B, C, D, E, and F (a reformatted reprint from Baumgärtel et al., 2015b).

Figure 2 shows the cafeteria scenario used for measuring the HRIRs which were used to simulate all scenarios measured in this study. In total, the HRIR database provided 12 HRIRs. Six different sound sources (loudspeaker symbols) were placed in the cafeteria (labeled A to F), with two listener orientations in which the listener would be facing either Sound Source A or D. In this study, the virtual listener was facing Sound Source A as shown by the head symbol, and the corresponding six HRIRs were used to simulate the sound sources. Speaker A was in front of the listener 102 cm away. The speaker at Position C was located 52 cm to the left of the listener but was directed toward Position A. The speaker at Position D was at a height of 151 cm and is to the right of the listener at 162 cm. Speakers B, E, and F were 117.5 cm, 129 cm, and 131 cm away from the listener, respectively. The speakers at Positions A, B, C, E, and F were positioned at a height of 111 cm.

Note that the simulated cafeteria scenario was not symmetric with respect to the left and right ear due to the different distance of the speakers and the close reflective surface on the left side. In order not to intermingle side-specific effects of the bimodal CI users with side-specific effects of the acoustic scenario, in this study, only CI users with a CI on the same side (here: right) were recruited.

### CI Fitting

Before testing, subjects were provided with a loaner CP910 sound processor to be used as the study sound processor during testing. Their favorite everyday map on their own sound processor was copied onto the study processor. On this processor, the signal processing algorithms Beam, Zoom, ASC, SNR-NR, WNR, and ADRO available in the CP910 were switched off. In addition, DAI was enabled, and the microphones were disabled in the processor by setting the Accessory Mixing Ratio to “Accessory only.”

### Presentation Setup

**Figure 3.**
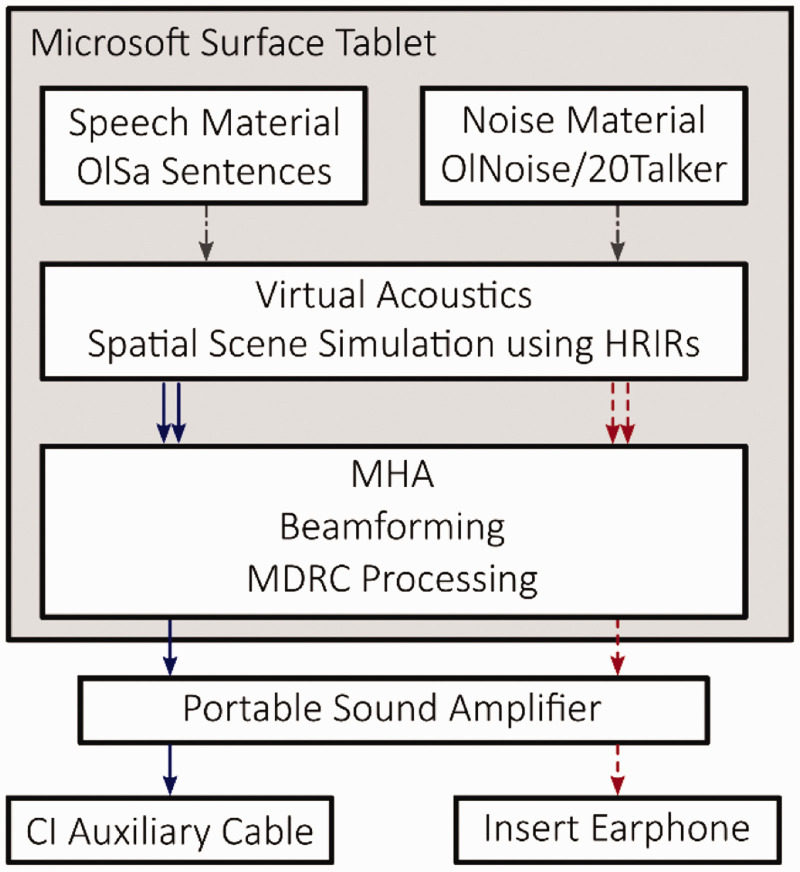
Block Diagram of the Presentation Setup. Blue solid lines represent the right (CI) side audio signals, while red dashed lines represent left (HA) side audio signals. The black dash-dotted line represents single-channel signals. Signal convolution with HRIR, speech, and noise mixing was done using MATLAB, followed by beamforming and multiband dynamic range compression usually performed in HA, and CI sound processor devices were emulated using the MHA software. The output of the Microsoft Surface tablet was then connected to a portable sound amplifier. Finally, the right channel output of the portable sound amplifier is connected to the CI sound processor via an audio cable, and the left channel output is delivered to the subject using an insert earphone. OlSa = Oldenburg sentence test; HRIR = head-related impulse responses; MHA = master hearing aid; MDRC = multiband dynamic range compressor; CI = cochlear implant.

Figure 3 shows the stimulation setup used in this study. Speech intelligibility tests were conducted using a Windows Surface tablet connected to a portable sound amplifier (FiiO E12, cross-feed, gain and bass switches were set to off), which was used to amplify the audio signals for the HA (simulated using the MHA) and the CI. The study CI processor was connected to the amplifier via Cochlear’s auxiliary input cable. An insert earphone connected to the amplifier was used to present the simulated HA signal to the subject’s contralateral ear. To account for typical processing delays of HAs with respect to a CI (Zirn et al., 2015), a delay of 6 ms was introduced into the HA simulation path after the beamformer and dynamic range compressor. The MHA was run on the tablet emulating both beamformers, ADM and MVDR, as well as a multiband dynamic compression algorithm. The gain applied to compensate for the subjects’ hearing loss was prescribed according to CAMFIT (Moore et al., 1999). CAMFIT focuses on amplifying low frequencies and limits amplification to 5 kHz. In agreement with Williges et al. (2019), it was modified to limit amplification to 105 dB SPL (Haumann et al., 2012) and to avoid amplification in frequency regions where the listener had a hearing loss of more than 90 dB HL (Zhang et al., 2014).

### Preprocessing Algorithms

This section describes the two beamformer algorithms that were used in this study, the ADM and the MVDR. Both algorithms were provided through the MHA and had the same implementation as in Baumgärtel et al. (2015a, 2015b) and Völker et al. (2015).

The ADM beamformer was used as a monaural beamformer (Elko & Pong, 1995) and implemented independently on each side (HA and CI). The ADM used the signals of two omnidirectional microphones on the HA casing or CI sound processor, which in this case were the simulated microphone signals, to generate a monaural denoised signal using spatial filtering. The distance between the front and rear microphones simulated in this study was 14.9 mm as specified in Kayser et al. (2009). This distance may vary between different devices used by HA and CI users and will affect the performance of beamformers (Bitzer et al., 1999; Dillon, 2012). The ADM started by generating two fixed cardioids with one of them pointing to the front and the other to the back. Then, the frontal cardioid was added to the back-pointing cardioid that was multiplied by a weighting factor β. The value of β was updated in an adaptive procedure that aimed at minimizing the energy at the output of the mixture. This procedure aimed at muting the loudest noise source behind the user. Generally, ADMs are particularly suitable for application in scenarios with a single noise source, especially when the noise source originated in the rear hemisphere. This also implies that the ADM required time to adapt to the noise signal. Hence, during speech intelligibility assessment, the signals were prepended with 3 s of noise before presentation of the speech and noise mixture to allow the ADM to adapt.

The MVDR (Doclo et al., 2015; Van Veen & Buckley, 1988) was used as a fixed binaurally implemented beamformer. The MVDR was designed around keeping the target signal (assumed to be in front of the listener), that is, speech, undistorted while suppressing noise. The prefix “fixed” refers to the fact that the MVDR has a fixed beam profile and does not steer a spatial zero toward noise sources as the ADM algorithm does. Instead, the MVDR used the four-microphone inputs with two placed on each side of the listener to produce spatially filtered left and right signals. In other words, the filter coefficients of the MVDR,$\;W_{L}$ and${\;W}_{R}$, can be calculated beforehand and saved on the HA or CI device, as was done in the MHA implementation used in this study. To achieve that, several variables were required for that calculation. It required a spatial coherence matrix Γ that was designed assuming a spatially diffused noise. Moreover, it needed the anechoic head-related transfer functions (HRTFs) of the four microphones, combined in matrix A. In addition, it also required the anechoic HRTF of the front microphone of the left and right devices, denoted as$\;A_{L}$ and${\;A}_{R}$, respectively. Then, the frequency-domain filter coefficients, $W_{L}$ and${\;W}_{R}$, were calculated as follows:

$$W_{L}=\frac{\Gamma^{-1}A}{A^{H}\Gamma^{-1}A}{A^{*}}_{L}^{\;}$$

$$W_{R}=\frac{\Gamma^{-1}A}{A^{H}\Gamma^{-1}A}{A^{*}}_{R}^{\;}$$

The four-microphone input signals from the left and right sides within each scenario were then multiplied with $W_{L}$ and $W_{R}$ to produce the left and right (enhanced) output signals, respectively. This required synchronized access to the four-microphone input signals. It was simple to provide synchronized signals in this study because all algorithms were simulated in one computer, and output signals were transferred to the subject via DAI cable and insert earphone. However, an application in real life would require a link between the two devices.

The MVDR was shown to be superior to the ADM in its noise suppression performance (Baumgärtel et al., 2015b). This is due to the higher number of microphones and larger spatial separation between their positions as explained in Bitzer et al. (1999) and Chapter 7 of Dillon (2012), thus leading to a sharper spatial beamformer directivity even at lower frequencies. The MVDR was designed assuming a diffuse noise field that is supposed to result in a superior performance compared with the ADM in diffuse acoustic conditions (Baumgärtel et al., 2015a). Furthermore, noise reduction algorithms in general do result in spectral and spatial cue distortions. That includes the ADM and MVDR beamformers. The term *distortionless* in the MVDR refers to the undistorted signal coming from the front of the listener. However, binaural cues based on sounds from other directions, which can be necessary for spatial perception, will still be distorted by the algorithm (Baumgärtel et al., 2015a), including speech signal reflections caused by room geometries, like the glass ceiling to the left side of the listener.

### Loudness Balancing

For each subject, loudness was balanced between CI and HA by presenting short broadband noise bursts first to the HA side and then to the CI side using the procedure described in Veugen et al. (2016). The noise was presented from Position A of the simulated cafeteria scenario with a duration of 1.5 s and an initial level of 65 dB SPL. The subject was asked to increase or decrease the level of the noise burst on the CI side to match the loudness of the noise burst on the HA side. This was repeated until the subject judged the signal loudness on the CI side to be the same as for the signal on the HA side.

### Study Design

In addition to the three beamformer conditions, four different spatial settings were investigated. Speech was presented from the front (speaker at Position A) of the listener with a fixed level of 65 dB SPL. The noise used in the first, second, and third scenario was a stationary noise with long-term average speech spectrum (“OlNoise”), which was presented from Positions C (left side, S_0_N_–90_), A (front, S_0_N_0_), or D (right side, S_0_N_+90_), respectively. As all subjects used an HA on the left side, and a CI on the right side, the noise was facing the HA in the S_0_N_–90_ scenario and the CI in the S_0_N_+90_ scenario. Note that these two scenarios also differ in the distance of the noises, that is, the noise source is closer to the HA (52 cm) than it is to the CI (162 cm). In the fourth spatial scenario, labeled as S_0_N_20TB_, speech was presented in multitalker babble noise. The S_0_N_20TB_ was the same scenario measured in Baumgärtel et al. (2015a) and was created by convolving four soundtracks from the EUROM1 corpus (Chan et al., 1995) with each one of the sound source positions (B through F), adding to a total of 20 talkers. However, as only five tracks were available, each of the five soundtracks was reused four times.

Prior to testing, each subject was familiarized with the OlSa speech material by administering one list of 20 OlSa sentences in quiet with both noise and speech coming from the front, that is, S_0_N_0_. Afterward, one 20-sentence list of the OlSa in the S_0_N_0_ noise scenario was used for familiarization preceding the first measurement out of the S_0_N_–90_, S_0_N_0_, or S_0_N_+90_ scenarios. Furthermore, one 20-sentence OlSa list in the S_0_N_20TB_ noise scenario was used for training preceding the measurement within the S_0_N_20TB_ scenario. Each combination of algorithm condition and noise scenario was measured twice (test and retest). During testing, test and retest of speech recognition within one scenario were performed in direct sequence, whereas SRTs were assessed in random order across the four noise scenarios. All measurements were performed in two sessions of maximum 2 hr distributed over two consecutive days.

### Statistical Analysis

IBM SPSS Statistics version 25.0 (Armonk, NY, USA) was used for data analysis. The data were tested for normality using the Shapiro–Wilk test. Mauchly’s test was applied to check for the sphericity of the distribution of the SRTs. A three-way repeated measures analysis of variance with the factors test–retest, algorithm condition, and spatial scene was performed on the SRTs. To assess the effect of preprocessing condition and spatial scenario, a two-way repeated measures analysis of variance with the factors preprocessing strategy (NoBF, ADM, and MVDR) and spatial scenario (S_0_N_–90_, S_0_N_0_, S_0_N_+90_, and S_0_N_20TB_) was then conducted. Greenhouse–Geisser correction was used to modify the degrees of freedom of the factors violating sphericity. After statistically significant effects were found, Bonferroni-corrected post hoc analysis *t* tests were applied to compare and assess benefit in speech recognition as well as SRM in the different spatial scenarios with the different algorithms by analyzing one factor at a time. The algorithm benefit (B) was defined as the difference between the SRT obtained for NoBF and SRT with the ADM or MVDR, that is, B_ADM_ = SRT_NoBF_ – SRT_ADM_, and B_MVDR_ = SRT_NoBF_ – SRT_MVDR_. SRM was defined as the difference between the SRT for S_0_N_0_ and the SRT for presentation of noise at the side being evaluated, that was, SRM_+90_ = SRT(S_0_N_0_) – SRT(S_0_N_+90_), and SRM_–90_ = SRT(S_0_N_0_) – SRT(S_0_N_–90_).

## Results

The aim of this study was to investigate the influence of the ADM and MVDR on SRTs measured in bimodal CI users. This section presents the measured SRTs, algorithm benefit, and SRM, and their statistical analysis.

### Speech Reception Thresholds

An effect of test order, F(1,9) = 5.098, *p* = .034, η^2^ = 0.447, was observed; however, the test–retest difference in SRT of 0.25 dB was much lower than the 1 dB test–retest reliability of the OlSa sentence test (Wagener et al., 1999). Therefore, test and retest SRT measurements were averaged for each participant and test condition, and the averaged SRTs were used for further statistical testing.

**Figure 4.**
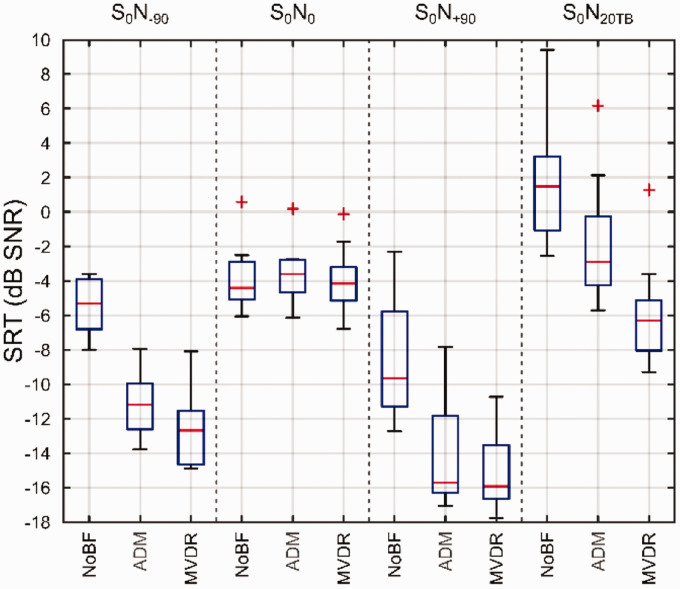
Box-Whisker Plots of SRTs of Nine Bimodal CI Listeners as a Function of the Spatial Scenario (S_0_N_–90_, S_0_N_0_, S_0_N_+90_, and S_0_N_20TB_) and the Preprocessing Condition (NoBF, ADM, and MVDR). Note that the noise faces the HA and CI in the S_0_N_–90_ and S_0_N_+90_ scenarios, respectively. Edges of the boxes mark the 25th and 75th percentiles, and whiskers extend to data points that are within 1.5 times of the interquartile range. SRTs outside of this range are marked as outliers by red plus signs. NoBF = no-beamforming; ADM = adaptive differential microphone; MVDR = minimum variance distortionless response; SRT = speech reception threshold; SNR = signal-to-noise ratio.

Figure 4 shows the SRTs, averaged across the two repetitions, of the nine bimodal CI listeners as box-whisker plots obtained for three preprocessing conditions, NoBF and the two beamformers ADM and MVDR, in each of the four spatial scenarios (S_0_N_–90_, S_0_N_0_, S_0_N_+90_, and S_0_N_20TB_). Lower (that is, more negative) SRTs denote better speech-in-noise performance. Averaged across spatial scenarios, the SRTs were significantly affected by the preprocessing condition, F(1.770,14.157) = 423.173, *p* < .001, η^2^ = 0.981. Likewise, there was also a significant effect of spatial scenario on the SRT, F(3,24) = 125.418, *p* < .001, η^2^ = 0.940. Moreover, there was a significant interaction between spatial scenario and the preprocessing condition, F(6,48) = 32.237, *p* < .001, η^2^ = 0.801.

### Algorithm Benefit

**Figure 5.**
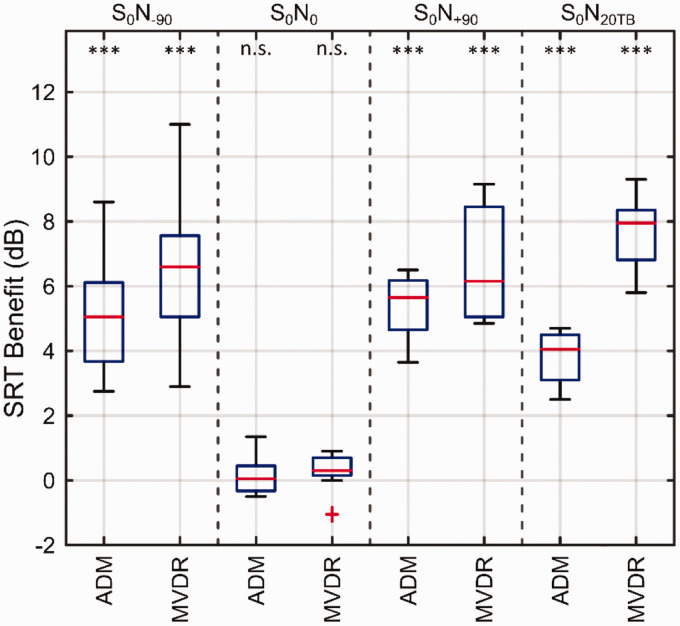
Box-Whisker Plots of SRT Benefits of Nine Bimodal CI Listeners Obtained with the Beamformers ADM and MVDR Compared With the NoBF Condition for Each of the Spatial Scenarios (S_0_N_–90_, S_0_N_0_, S_0_N_+90_, and S_0_N_20TB_). Note that the noise faces the HA and CI in the S_0_N_–90_, S_0_N_+90_ scenarios, respectively. Each panel shows the SRT improvements with both noise reduction algorithms, ADM and MVDR, for one of the spatial scenarios. For both algorithms ADM and MVDR, and each spatial scenario, asterisks denote the statistical significance of algorithm benefit versus the NoBF condition as being different from zero dB. ***p < .001. ADM = adaptive differential microphone; MVDR = minimum variance distortionless response; SRT = speech reception threshold; n.s. = not significant.

For each spatial scenario, pairwise Bonferroni-corrected post hoc *t* tests were applied to reveal significant differences in SRT between the preprocessing conditions. Figure 5 shows box-whisker plots of the SRT improvements achieved by using the ADM or MVDR compared with the NoBF condition for the spatial scenarios S_0_N_−90_, S_0_N_0_, S_0_N_+90_, and S_0_N_20TB_.

As shown in Figure 5, there was a significant large benefit in SRT for the application of the ADM as well as the MVDR between 3.5 and 7.5 dB in all spatial scenarios except S_0_N_0_.

Moreover, compared with the ADM, the MVDR allowed for lower SRTs, that is, better speech-in-noise performance, in the S_0_N_–90_ and S_0_N_20TB_ scenarios. Table 2 summarizes the results of the pairwise post hoc comparisons of SRTs between preprocessing conditions for each of the spatial scenarios. The MVDR provided higher SRT benefit in S_0_N_–90_ (1.5 dB, *p* < .001), and there was an even larger difference in benefit in S_0_N_20TB_ (4.0 dB, *p* < .001).

**Table 2.** Group Median SRT Benefits (in dB) Obtained as Medians of Pairwise Differences in SRT Between the Preprocessing Conditions Indicated in the Row Labels for Each of the Four Different Spatial Scenarios.

Algorithm/Spatial scenario	S_0_N_–90_	S_0_N_0_	S_0_N_+90_	S_0_N_20TB_	Overall
ADM vs. NoBF	5.5***	−0.2	5.4***	3.5***	3.6***
MVDR vs. NoBF	7.0***	0.4	6.4***	7.5***	5.3***
MVDR vs. ADM	1.5***	0.2	1.0	4.0***	1.7***

*Note*. ADM = adaptive differential microphone; NoBF = no-beamforming; MVDR = minimum variance distortionless response.

***p < .001.

### Spatial Release From Masking

**Figure 6.**
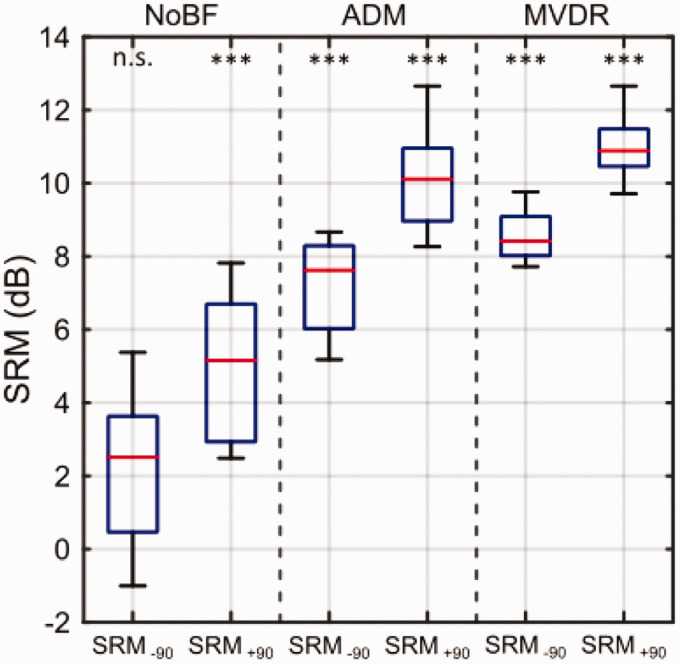
Box-Whisker Plots of SRM of Nine Bimodal CI Listeners for the Preprocessing Conditions NoBF, ADM and MVDR for the Spatial Scenarios S_0_N_–90_ (Noise Facing the HA) and S_0_N_+90_ (Noise Facing the CI), Indicated by SRM_–90_ and SRM_+90_. For each algorithm and each SRM, asterisks denote the statistical significance of SRM as being different from zero dB. ***p < .001. NoBF = no-beamforming; ADM = adaptive differential microphone; MVDR = minimum variance distortionless response; n.s. = not significant; SRM = spatial release from masking.

Figure 6 shows box-whisker plots of individually extracted SRMs for the three preprocessing conditions, NoBF, ADM, and MVDR, depending on the noise direction. SRM_–90_ refers to the SRM achieved when calculating SRM for noise from –90°, and SRM_+90_ refers to the SRM achieved when calculating SRM for noise from 90°. For the NoBF condition, Bonferroni-corrected pairwise comparisons of SRT showed a nonsignificant SRM of 2.0 dB (*p* = .114) once the noise is moved to the left side (–90°) when compared with the frontal noise condition (S_0_N_0_), and a significant SRM of 5.2 dB (*p* < .001) when the noise is moved to the right side (+90°) of the subject. Moreover, the bimodal CI subjects showed SRMs of 7.3 dB (*p* < .001) or 10.4 dB (*p* < .001) with ADM and 8.7 dB (*p* < .001) or 11.2 dB (*p* < .001) with MVDR, when the noise is moved to the left or right side, respectively.

There was a significant spatial release of masking in all preprocessing conditions with regard to both SRM_–90_ and SRM_+90_, except for SRM_–90_ in the NoBF condition.

## Discussion

This study investigated spatial speech-in-noise performance in bimodal CI users for two-directional preprocessing algorithms while controlling for head movements, CI sound processor and HA processing and fitting in a simulated realistic cafeteria environment. To avoid head movements as an interfering factor, the present study controlled for head movements by using a HRIR database for virtual acoustics (Kayser et al., 2009) to generate the desired spatial scenario and presented the signals via DAI cable and insert earphone. Using the independent, but bilaterally implemented ADM resulted in significant improvements in SRT for both single noise source scenarios and the 20-talker babble diffuse noise scenario. Moreover, the MVDR—using the four microphone signals from both sides jointly—resulted in a significantly higher improvement (1.7 dB, *p* < .001) averaged across all spatial scenarios compared with the ADM that utilizes the two microphone signals from either side separately. As expected, there was no benefit in SRT, neither with ADM nor MVDR, in the S_0_N_0_ scenario as spatial separation of speech and noise did not exist to allow the algorithms to reduce the noise level.

Baumgärtel et al. (2015a) assessed the SRT benefit with the ADM and MVDR in bilateral CI users using the same 20-talker babble in the same virtual acoustics cafeteria scenario (among other spatial scenarios) and found benefits with the ADM and MVDR of 3.5 dB and 6.9 dB, respectively, which are similar to the 3.5 dB and 7.5 dB obtained in this study. Völker et al. (2015) reported a similar SRT benefit of 4.1 dB in the same 20-talker babble scenario with bilateral HA users and normal-hearing listeners when using the ADM. However, they found a considerably lower SRT benefit of only 4.3 dB in these subjects using the MVDR compared with the benefit revealed by Baumgärtel et al. (2015a) and the present study. As explained by Baumgärtel et al. (2015a), the SNR improvement is therefore partially outweighed by the side effect of binaural cue distortion which is used in the normal-hearing auditory system to spatially isolate target speech/signals. However, this mechanism is not present in bilateral and bimodal CI users, due to the inability of the CI to transmit the acoustic fine structure (see, e.g., Wilson et al., 1991). Therefore, like bilateral CI users, bimodal CI users are one target group where binaurally implemented beamformers, such as the MVDR, have a high potential to provide better speech-in-noise performance.

SRT benefits obtained with similar spatial noise reduction algorithms in bimodal CI users were also found in studies in which speech and noise were presented in free field using loudspeakers in a low reverberant room or sound attenuated booths (Buechner et al., 2014; Devocht et al., 2016; Ernst et al., 2019; Mosnier et al., 2017). The ADM implementation used in the present study was comparable to the implementation of Sonova’s monaural adaptive beamformer, UltraZoom, as it used the same theoretical basis described in Elko and Pong (1995), as indicated in Buechner et al. (2014). UltraZoom is implemented in the CI sound processors Naída CI Q70 and Naída CI Q90 and the HA Naída Link (Sonova, Stäfa, Switzerland). Both the fixed MVDR used in this study and the StereoZoom available with bilateral and bimodal Naída CI and HA devices are binaural beamformer algorithms and use a bilateral exchange of audio signals for spatial filtering. However, their implementation cannot be compared directly, due to lack of knowledge regarding implementation details of StereoZoom.

As stated in the Introduction section, head movements can influence the performance of beamformer algorithms. Head movements will change the SNR at each ear (Gifford et al., 2015), which might have influenced prior studies conducted in free field (Buechner et al., 2014; Ernst et al., 2019; Vroegop et al., 2018; Weissgerber et al., 2017). Moreover, when moving the head, adaptive beamformers need time to adjust to the new target direction. Hendrikse et al. (2020) found this effect of algorithm adaptation to be less than 1 dB in realistic everyday scenarios comparable to ours. They also investigated the extent to which the SNR improvement provided by beamformers changes during head movements in realistic scenarios. They traced the head movements of subjects listening to an audiovisual speech task in a realistic scenario and used the traces to recreate the speech and noise signals at the subjects HA microphones. Afterward, they measured the SNR before and after applying the beamformers on the signals in 200 ms blocks of time to calculate the SNR improvement for each of the time blocks and each subject. The measured differences in improvement were considerably large, sometimes reaching up to 15 dB. Furthermore, Ernst et al. (2019) investigated the effect of subject-specific HRTFs versus KEMAR HRTFs and found a reduction of algorithm benefit, when using subject-specific HRTFs (via T-Mics). Taken together, these results suggest a benefit of beamforming algorithms, even under less controlled conditions in everyday life.

Compared with NoBF, the 3.5 dB benefit achieved with ADM in S_0_N_20TB_ in the present study is consistent with SRT benefits in bimodal CI users using UltraZoom. Buechner et al. (2014) reported a benefit of 5.3 dB obtained in a low reverberant room with a frontal speaker for speech and speakers at ±70°, ±135°, and 180° for noise (OlNoise) presentation. Devocht et al. (2016) reported an SRT benefit of 2.6 dB when speech was presented from the front, and noise speakers were arranged around the listener at angles of ±45°, ±90°, and 180°. They used stationary and fluctuating noises. Ernst et al. (2019) showed that changing the position of five noise-presenting speakers from {±60°, ±120°, and 180°} to {±30°, ±60°, and 180°}, that is, moving these speakers closer to the frontal target speaker, reduced the benefit with UltraZoom from 3.4 dB to 1.4 dB and diminished the SRT benefit with StereoZoom from 4.6 dB to 2.6 dB in bimodal CI users. They also reported a similar trend for bilateral CI users, where the SRT benefit with UltraZoom and StereoZoom was reduced from 4.3 dB to 1.8 dB, and 5.2 dB to 3.4 dB, respectively. Consistent with their SRT benefits with UltraZoom for the reduced separation of speech and noise sources, Weissgerber et al. (2017) reported a relatively small SRT benefit of 0.9 dB for bimodal users using the fixed monaural beamformer algorithm Zoom and the adaptive monaural Beam (both from Cochlear, Sydney, Australia) with noise presented from ±28.6° and ±151.4°. The large effect of the arrangement of noise sources on SRT benefit may explain the differences in SRT benefit across these four studies. However, other factors such as the beamformer algorithm used, noise types, and room acoustics may play a role in that difference as well. Because these studies were performed in free field, there was a limited ability to control for head movements. However, the head-movement-induced change in SNR algorithm benefit may be another explanation for the differences of SRT benefit across studies. As the present study controlled for head movements and provided the ability to study the spatial benefit of spatial speech enhancement algorithms without interference of this factor, slight differences to other studies evaluating monaural and binaural beamformers were observed that did not follow similar procedures to control for these factors. However, those differences cannot be solely attributed to head movements specifically due to different test conditions and study designs.

The results of this study showed that the bimodal CI subjects performed poorest in the S_0_N_20TB_ scenario, that is, had the highest SRTs in this scenario, which used a noise that was more diffuse and contained stronger fluctuations than in the other scenarios. This relates well to the findings of Devocht et al. (2016) and Weissgerber et al. (2017) who showed that bimodal CI listeners have poorer performance in noises with stronger fluctuations. Compared with NoBF, the MVDR resulted in a 7.6 dB SRT improvement in the S_0_N_20TB_ spatial scenario that was consistent with the 7.1 dB improvement of SRT with StereoZoom in bimodal CI users shown by Buechner et al. (2014). In addition, the SRT benefit with MVDR was similar across the different spatial scenarios S_0_N_–90_ (7.0 dB), S_0_N_+90_ (6.5 dB), and S_0_N_20TB_ (7.5 dB). However, following the same comparison, the SRT benefit of using the ADM was noticeably lower in the S_0_N_20TB_ (3.5 dB) scenario, compared with S_0_N_–90_ (5.5 dB) and S_0_N_+90_ (5.4 dB). Moreover, the SRT benefit with the MVDR was significantly higher than ADM in all spatial scenarios except S_0_N_0_. There are several properties of the MVDR that may explain its better performance:

- The MVDR used the four microphones jointly and hence can make effective use of the large physical separation between them which provides an acoustic advantage in comparison to the ADM that uses separate pairs of closely spaced microphones on either side of the head with small distance in between them (Bitzer et al., 1999; Dillon, 2012).
- The MVDR was specifically designed to reduce diffuse noise (Doclo et al., 2015) that is present in multisource and reverberant environments. Note that the simulated cafeteria scenario here was highly reverberant (T60 = 1.25 s; Kayser et al., 2009), which resulted in reverberation also in the single-source noise scenarios, for example, S_0_N_–90_ and S_0_N_+90_. Moreover, the difference in benefit for the MVDR versus ADM was more pronounced in S_0_N_20TB_ compared with S_0_N_–90_ and S_0_N_+90_ scenarios, that is, the S_0_N_20TB_ scenario exhibited more diffusiveness as several interfering talkers were distributed across the auditory scene, thus better representing the MVDR processing assumptions.

This study measured bimodal CI listeners in a scenario involving two asymmetries, one was that all participants had their CI on the right side and HA on the left side. The second asymmetry involved the noise sources: The scenario with noise from left (corresponding to S_0_N_−90_) had the noise source closer to the listener than the scenario with noise from right (corresponding to S_0_N_+90_). Disentangling the effects of the acoustic scenario from the effects of the device would require a larger number of participants including subjects with a CI on the left side and HA on the right side, which was not the intention of the present study. The SRM results (see Figure 6) showed for all three preprocessing conditions (NoBF, ADM, MVDR) higher SRM values for SRM_+90_ (noise from right, noise farther away) than for SRM_–90_ (noise from left, noise closer). If the distance of the noise source was the main factor leading to changes in SRM, an opposite effect would be expected: The closer the noise source, the higher the SRM (Rennies et al., 2011). Therefore, the asymmetry in SRM found here is most likely caused by the fact that most subjects’ poorer performing ear (when tested in isolation) was the CI on the right side: Moving the interferer from the front to the poorer side (here noise to right side) increases the SNR for the better ear (for most subjects the HA side) and thus provides a higher benefit than for the opposing situation.

Most likely, the deliberately introduced 6 ms delay in the HA path which is used to account for latency differences between CI and HA did not have a strong influence on the measured SRTs. As the delay was imposed on the HA path after the preprocessing algorithms, the output of both the ADM and the MVDR was not affected by it. The delay may, however, have affected the patient’s access to interaural time difference (ITD) information because it may transfer ITDs outside of the physiologically plausible range (about 0.66 ms maximum). This is known to affect localization ability in normal-hearing and bimodal CI users (Zirn et al., 2019). Although the effect on speech-in-noise performance has, to the author’s knowledge, not been tested yet in bimodal CI users, significant effects are very unlikely, because the task-specific better-ear-listening in bimodal CI users (Williges et al., 2019) does not require access to ITDs (Zedan et al. 2018).

Taken together, this study focused on benefits in speech intelligibility in noise in bimodal CI users for application of two beamformer algorithms, ADM and MVDR. Nevertheless, it did not evaluate the subjective quality of speech, which may be an interesting and important issue to investigate in future studies.

## Conclusions

SRTs of bimodal CI users were measured in four spatial scenarios with three preprocessing conditions. The “no beamformer” (NoBF) condition was compared with the adaptive directional microphone (ADM), a monaural noise reduction algorithm implemented independently on both sides of the listener, and the MVDR, a binaural noise reduction algorithm.

- The findings indicate a large and significant benefit in SRT with both algorithms and thus confirm earlier evidence of spatial noise reduction algorithms in bimodal CI users. Neither of the algorithms resulted in an improvement in the colocated speech and noise scenario. However, they also did not result in a degradation in SRT as well.
- The controlled configuration of the study, that is, the application of a simulated reverberant cafeteria scenario with different realistic spatial interferer configurations, eliminated a potential effect of head movements, and as all subjects used the CI on the right ear, a potential effect of implantation side on the algorithm benefit.
- The MVDR yielded larger improvements in SRT (1.0 dB to 4.0 dB higher) compared with the ADM that ranged from slight to considerable SRT benefits depending on the spatial scenario. This clearly shows the additional benefit of using a binaural beamformer compared with independently operating monaural beamformers.
- The largest difference in SRT between the MVDR and ADM was observed in the S_0_N_20TB_ scenario which included both reverberation and diffuse noise sources. This advocates for the usage of the MVDR in such acoustically difficult situations. However, the additional costs for providing a binaural link across hearing devices on both sides (e.g., higher energy consumption and latency issues) is still a considerable factor that must be weighed against the benefit achievable with this binaural beamformer algorithm.
- The results presented here encourage the usage of the ADM in everyday life scenarios for bimodal CI users. Truly binaural beamforming algorithms should see wider implementation for bimodally aided subjects. The MVDR was shown to be especially beneficial for bimodal CI users compared with bilateral HA users who have access to binaural processing abilities. Notably, this holds for rather complex and reverberant spatial conditions comparable to the S_0_N_20TB_ condition employed here.
